# Investigation the effect of water addition on intermolecular interactions of fatty acids-based deep eutectic solvents by molecular dynamics simulations

**DOI:** 10.1038/s41598-023-33234-8

**Published:** 2023-05-08

**Authors:** Samaneh Barani pour, Jaber Jahanbin Sardroodi, Alireza Rastkar Ebrahimzadeh, Gholamreza Pazuki

**Affiliations:** 1 Molecular Science and Engineering Research Group (MSERG), Molecular Simulation Lab, Azarbaijan Shahid Madani University, Tabriz, Iran; 2 Molecular Science and Engineering Research Group (MSERG), Department of Chemistry, Molecular Simulation Lab, Azarbaijan Shahid Madani University, Tabriz, Iran; 3 Molecular Science and Engineering Research Group (MSERG, Department of Physics, Molecular Simulation Lab, Azarbaijan Shahid Madani University, Tabriz, Iran; 4 Department of Chemical Engineering, Amirkabir University of Technology, Tehran, Iran

**Keywords:** Computational biology and bioinformatics, Mathematics and computing

## Abstract

In this work, we focused on the interaction between hydrogen bond acceptor (HBA) and hydrogen bond doner (HBD) in the binary mixtures. The results showed that Cl^−^ anion plays a key role in the formation of DESs. Also, the structural stability of deep eutectic solvents based on fatty acids (FAs) and choline chloride (Ch^+^Cl^−^) at different ratios was investigated in water using molecular dynamics simulations. We observed that the interaction between the chloride anion and the hydroxyl group of the cation leads to the transition of HBA to the water-rich phase. These atomic sites have important rule in the stability of the eutectic mixtures based on FAs and Cl^−^ anion. However, it seems that the binary mixtures with the mole percent at 30% of [Ch^+^Cl^−^] and 70% of FAs have more stability than other ratios.

## Introduction

Organic solvents are frequently used in the chemical industries. These solvents are used to dissolve, extract other materials and production of products such as detergents, pesticides and many other products. Many organic solvents are recognized as carcinogens and expensive materials. Deep eutectic solvents (DESs) are attracting widespread technological interest as a low-cost alternative to conventional organic solvents^1^. DESs are widely used in many fields such as dissolution and separation, electrochemistry, and materials preparation^2^. Deep eutectic solvents are binary mixtures that have a large depression of melting temperature at the eutectic point relative to the melting temperature of the pure components^3^. Most of the deep eutectic solvents proposed so far are a combination of a hydrogen bond donor (HBD) and a hydrogen bond acceptor (HBA) at a well-defined stoichiometric proportion. The formation of hydrogen bonding between HBA and HBD leads to the reduction of the melting temperature of the binary mixtures^4^. DESs are prepared from raw materials of natural origin such as amino acids, sugars, organic acids, alcohols and etc.^4^. Deep eutectic solvents are as diverse fields of application of solvents such as ionic liquids (ILs). ILs are the recommended first option for eco-friendly processes within the framework of green chemistry. ILs are considered as the best green solvents for various kinds of reaction such as alkylation, polymerization and preextractions, etc.^5^. Probably the most important feature of ILs is its inability the formation of a regular crystal network dut to the geometrical asymmetry of the cations^5^. These solvents have melting temperature falls or below ≤ 100 °C^6^. However, Compared to ILs, deep eutectic solvents have advantages such as high thermal and chemical stability, being cheaper to make, much less toxic, and easy preparation methods. In 2003, deep eutectic solvents (DESs) based on choline chloride and urea were reported by Abbott et al.^7^. The ability to form hydrogen bond between urea and choline chloride leads to the largest depression in freezing point of the binary mixture at a urea: choline chloride 2:1 mol ratio.

The stability of DES in aqueous solutions makes them suitable for applications in different industries. The interaction between the species with water somewhat disrupts the interaction between hydrogen bond donors and acceptors in hydrophilic DESs. However, since these solvents have low toxicity, a feature that makes them suitable for the pharmaceutical industry. Despite various applications of hydrophilic DESs (high miscibility), this feature limits the utility of hydrophilic DESs for polar solutions^8^. However, DES-based on hydrophilic compounds have unique benefit such as the desulfurization of liquid fuel and natural gas and synthesis of nanomaterials, and electrochemistry. In the design of hydrophilic solvents utilizes choline chloride salt and quaternary ammonium salts (QAS) as HBA.

However, the length of the alkyl chain components of DESs could play a crucial role in the design of stable hydrophobic solvents. Hydrophobic deep eutectic solvents were introduced by van Osch and co-workers in 2015^8^. Hydrophobic DESs have a lot more efficiency in the removal of pollutants from aqueous. Accordingly, van Osch and co-workers showed that HDES-based quaternary ammonium salts and decanoic acid (DecA) have high extraction yields in the separation of volatile organic compounds^9^.

There is great research to investigate the stability of DESs in water by using MD simulation. A molecular-level understanding DESs in the presence of water and further investigations are necessary in this direction. Further study by the authors of this work was done in order to investigate the relationship between the percentage composition of the two components of HBA and HBD and the stability of DES in adjacency water. Partial disruption of the structural properties of the DESs occurs when DES is soluble in water. The fatty acid and choline chloride-based DES integrity is lost to some extent in adjacency water. The addition of the water molecules to box simulation containing deep eutectic solvents generally causes the dynamics to become fast. In this work, through molecular dynamics simulations, the influence of water on the dynamic and structural properties of DESs based on fatty acids (Caprylic acid, Lauric acid) and choline chloride were investigated in the adjacent water. The binary mixtures at different molar ratios of Ch^+^ Cl^−^ and acid were simulated in the adjacent water and the pure state at 353 K. In order to check the stability of DESs in the adjacent water, the distribution of two-component of DESs around each other was investigated by calculating the combined distribution functions (CDFs), radial distribution functions (RDFs), and angular distribution functions (ADFs), and spatial distribution functions (SDF). Furthermore, the dynamics of DESs is characterized by computing the self-diffusion coefficients, the mean square displacements (MSDs) and velocity autocorrelation functions (VACFs) of the center of mass (COM) of species as a function of time $t$. We find that the binary mixtures consisting of DES1 (Caprylic acid: Choline chloride) and DES2 (Lauric acid: Choline chloride) have stability in adjacency water.

## Methodology

**Figure 1:**
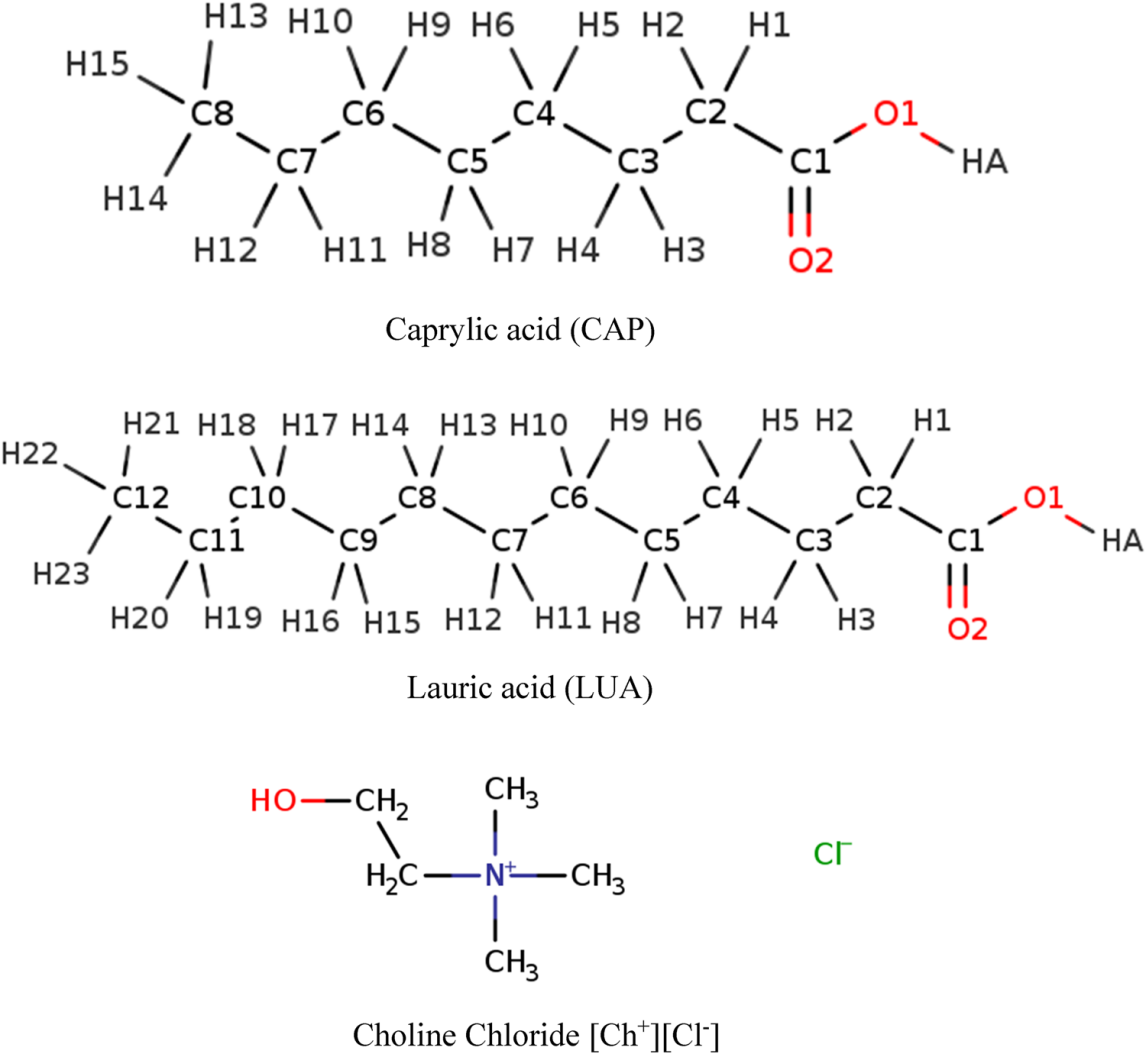
Schematic of choline chloride [Ch^+^][Cl^−^] and Fatty acids (FAs) with the main labels.

Initially, the structures of eutectic solvent componentes and pollutant molecules were drawn in Visual Molecular Dynamics (VMD) software. Then structures were optimized at the MP2/6-31G* theory level using the Gaussian 09 software^10^. Force field parameters and partial charges of species were obtained by fitting results from quantum-chemical calculations (More details about parameterization are given in the previous work)^11^. The results of this paper present that the shear viscosities obtained for deep eutectic solvents based on caprylic acid and thymol at a ratio of 1:1 using MD simulation are close to the experimental value, with a difference of less than 8.82%^11^. To preparation of initial simulation boxes, packmol-16.343.3 package were employed (The pachage is distributed as free software and can be downloaded from http://www.ime.unicamp.br)^12^. In the beginning, the cubic box containing 700 choline chloride ion pair and 300 fatty acid was randomly provided. Then, the mixtures are composed of species with the composition of fatty acid/choline chloride being 500: 500 and 700:300. Periodic boundary conditions were imposed in all directions to mimic bulk properties. Molecular dynamics simulation for all the studied systems was performed with time step 1 fs using the NAMD-2.12 package (https://www.ks.uiuc.edu/)^13^. The information of the simulated boxes and the different fatty acids and choline chloride salt with abbreviations are listed in Table 1. The structure of the compounds of the binary mixtures is shown in Fig. 1. First, the 5,000,000-step energy minimization was undertaken to remove bad van der Waals contacts of the binary mixtures. The binary mixtures were heated to a temperature of 353 K. All binary mixtures were equilibrated via a 50 ns in the NPT ensemble. The Langevin piston Nose–Hoover method and Langevin dynamics were applied to keep at constant temperature of 353 K and pressure, respectively. The equations of motion were solved by using the standard Verlet algorithm with a time step of 1 fs. The particle-mesh Ewald (PME) algorithm was used to calculate electrostatic interactions and a cutoff of 12 Å was set for Lennard–Jones interactions^14^. After the system reaches equilibrium, the dynamic and transport properties were investigated and the last 1 ns of the simulations was considered to combine with the water box. The two cubic boxes were combined together to get the periodic cell that we will refer to this as the binary systems in the adjacent water. The binary systems in the adjacent water were investigated under the same conditions of pure state.

**Table 1:** Names of the simulated eutectic solvents and their different combinations with abbreviations, and the HBA: HBD ratios in the binary mixtures.

Name	HBD	HBA	%FAs	n HBA: n HBD
CCA (DES1)	Caprylic acid (CAP)	choline chloride [Ch^+^][Cl^−^]	30, 50, 70	300:700, 500:500, 700:300
CLA (DES2)	Lauric acid (LUA)	choline chloride [Ch^+^][Cl^−^]	30, 50, 70	300:700, 500:500, 700:300

### Radial distribution and combined distribution functions

**Figure 2:**
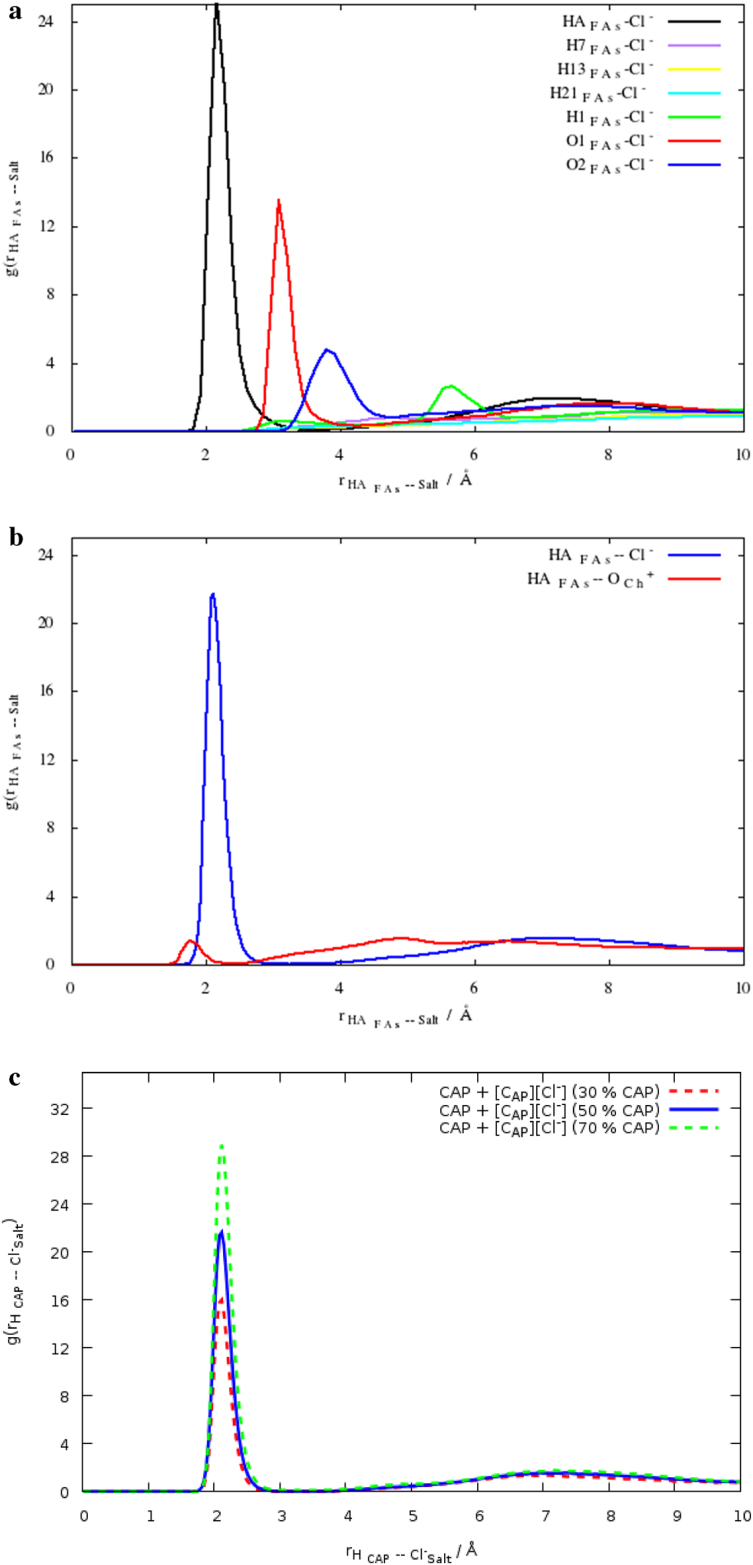

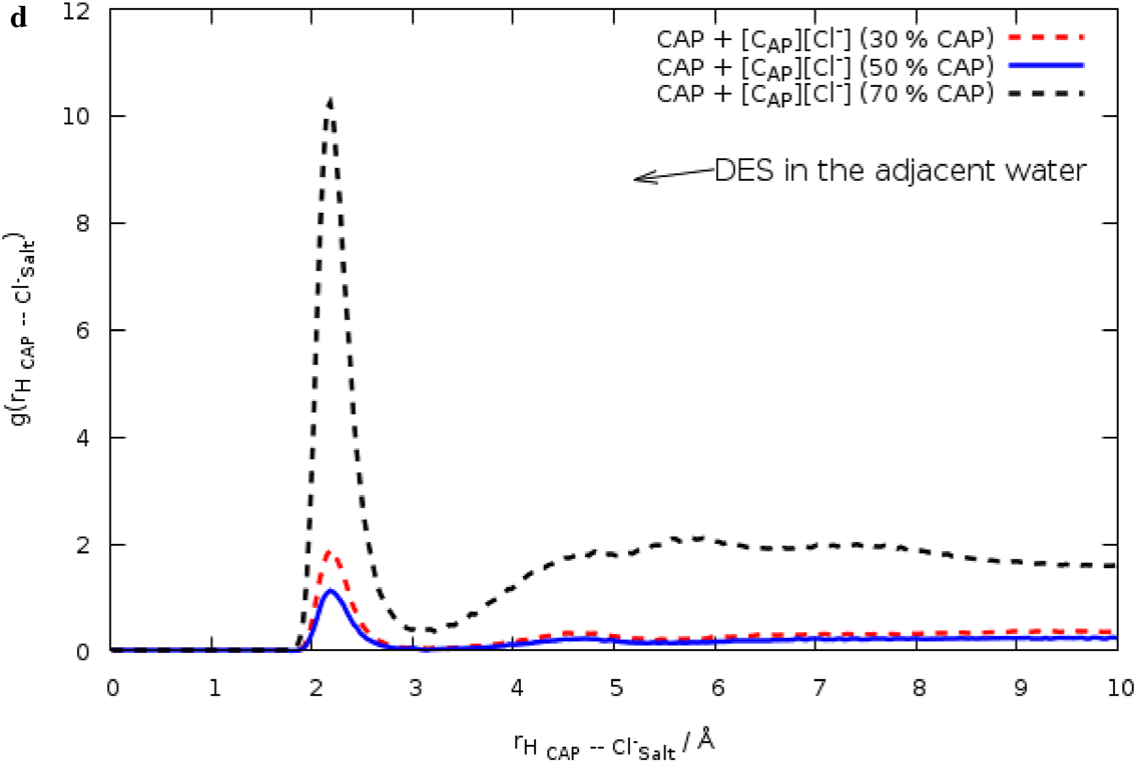
(**a**) Site–site RDFs between the different atoms of LUA and Cl^−^ anion for the binary mixtures with the mole percent at 50% at 353 K. (**b**) RDFs between CAP molecules and choline chloride salt, $g({r)}_{{Ch}^{+}-FAs}$, ${\mathrm{g}(\mathrm{r})}_{{Cl}^{-} -\mathrm{ FAs}}$, for the binary mixture with the mole percent of CAP at 50.0% at 353 K. (**c**) RDF between the HA atom of FAs and Cl^−^ anion for the binary mixtures with the mole percent 25%, 50% and 75% FAs at 353 K. (**d**) RDFs between the HA atom of CAP molecules and chloride anion,${\mathrm{g}(\mathrm{r})}_{{Cl}^{-} -\mathrm{ FAs}}$, for the binary mixture with the mole percent of CAP at 30%, 50.0% and 70% at the pure state and the adjacent water.

**Figure 3:**
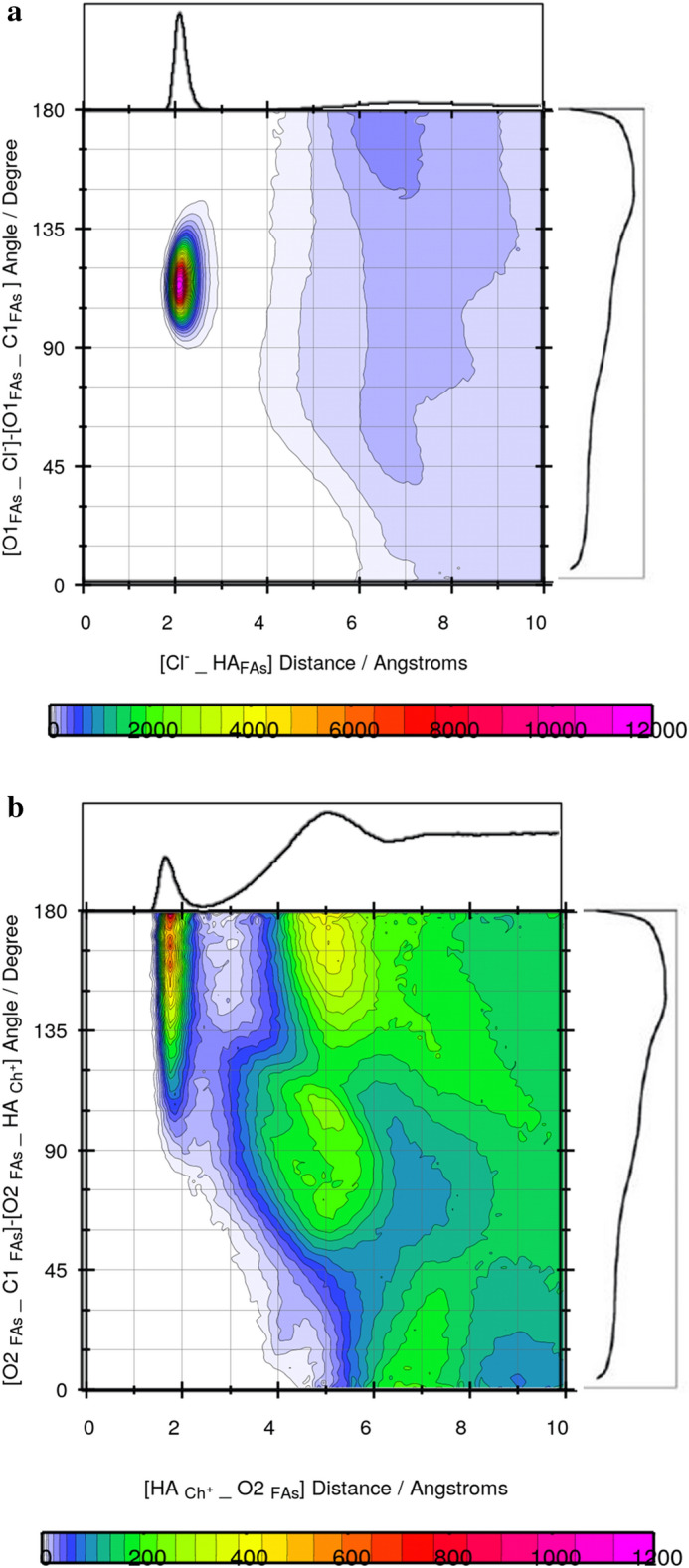
Combined radial/angular distribution functions for (**a**) the HA FAs _ Cl^−^ distance and C1CAP_O1CAP_Cl^−^ angle (**b**) the HA _Ch+_ _ O2 _FAs_ distance and C1_FAs_−O2 _FAs_−HA _Ch_^+^ angle.

The radial distribution function, g(r), and coordination numbers (CN) give information concerning the structural properties of the binary mixtures. The RDF, g(r), is related to the coordination number, or the number of neighbors, by the following equation:

1 $$CN = 4\pi {\int }_{0}^{{r}_{min1}}\rho g(r){r}^{2}dr$$

where the bulk density denoted by ρ. $g\left(r\right)$ is also called the pair distribution function and can describe the distribution of the species around any given specie in the system^15^. The site–site radial distribution functions (RDFs) have led researchers to examine in detail the structural properties. Therefore, special attention will be paid to site–site RDF analysis in this section. The different atomic sites of the species for drawing RDFs are the oxygen and hydrogen atoms of carboxyl (–COOH) of FAs and hydroxyl (OH–) functional groups of the Ch^+^ cations. This analysis can be useful for H-bond analysis, which will be examined in detail. It is the HA and OA atoms of FAs and the O and HA atoms of Ch^+^ cation. RDFs between HA atom of FAs and Cl^−^ anion in pure binary mixtures, as shown by ${g(r)}_{{Cl}^{-} - FAs}$, represnt a significantly sharp peak compared to other atoms of FAs at around 2.0 Å (see Fig. 2a). In addition, the peaks of all the RDF between the Cl^−^ and different atoms of HBD molecules appeared at long distances. Higher occurrence probabilities of H-bonds can be observed at 2.0 Å for the Cl^−^−HA_FAs_ interaction. Angular/distance probability region at 105°−120°/2.0−2.5 Å is a confirmation of the RDF results (see Fig. 3a). The structural correlations of HBA–HBD and the orientation of the individual solvent molecules were analyzed by combined distribution functions (CDFs). Angular Distribution Functions (ADF) were calculated using TRAVIS-200504 (This pachage can be downloaded from http://www.travis-analyzer.de/)^16^. ADFs of molecular dipole vectors are ontained from as:

2 $${ADF}_{abc}(\alpha )=\frac{1}{\mathrm{sin}(\alpha )}\frac{1}{{N}_{a}{N}_{b}{N}_{c}}\sum_{i=1}^{{N}_{a}}\sum_{j=i+1}^{{N}_{b}}\sum_{k=j+1}^{{N}_{c}} {\langle \delta (\alpha -\angle \left({\overrightarrow{r}}_{i}\left(t\right),{\overrightarrow{r}}_{j}\left(t\right),{\overrightarrow{r}}_{k}\left(t\right)\right))\rangle }_{t}$$

The position vectors can be used for specifying the angle of interest^17^. The preferred orientation was investigated by combining the RDF between the HA atom of [Ch^+^] and the O2 atom of FAs and an ADF between the C1 _FAs_−O2 _FAs_−HA _Ch+_ angle. Also, the angle between the C1–O1 vector in FAs and the intermolecular Cl^−^–O1 _FAs_ vector between anion and FAs can be defined for anion orientation around FAs. There is the maximum occurrence probability region at an angle range of 105−120° in the less distance ∼3 Å for anion and FAs interaction in the binary mixtures (see Fig. 3a). The maximum occurrence probability region was observed for Ch^+^cation and FAs at around 150°–180°/2 Å (see Fig. 3b). CDF analysis in the different ratios confirms that the orientation of anions around FAs is probable in the binary mixtures with the mole percent of CAP at 70.0%. It is clear from ${g(r)}_{{Cl}^{-} - FAs}$ combined with ADF analysis that Cl^−^ anions likely have a stronger hydrogen bonding interaction with the FAs molecules, and may form a weak hydrogen bond with cations in the binary mixtures (see Fig. S1a–c). The ${g\left(r\right)}_{{Cl}^{-}-\mathrm{ FAs}}$ and RDFs between choline cations and FAs represent the sharp peak at 2 Å, which the peak height in the ${(\mathrm{gr})}_{{Ch}^{+}-FAs}$ is less than ${\mathrm{g}(\mathrm{r})}_{{Cl}^{-} -\mathrm{ FAs}}$ (see Fig. 2b). It seems that the interaction between Cl^−^ anion, and HBD leads to a decrease in the strong correlation between anion and cation. To investigate the structural properties of the binary mixtures with the different ratio of FAs and Ch^+^/Cl^−^ salt, the RDFs was calculated at 353 K. For this purpose, the HA atom of FAs was chosen as a reference, and the distribution of the Cl^−^ anions around them was investigated. A significant interaction is observed between HBA and HBD in the binary mixtures with the mole percent of CAP at 70.0% (see Fig. 2c). The coordination number (CN) was calculated to the number of anions around FAs in the binary mixtures with different ratios (see Table S1). The coordination number can be obtained from the first peak of the RDF curve between species^18^. For ${g(r)}_{\mathrm{HBD }\_\mathrm{HBA}}$, the coordination number is 0.1871 in the binary mixture with the mole percent of CAP at 30.0%, whereas, CN of RDF between HBA and HBD in the binary mixture with the mole percent of CAP at 70.0%, is 0.6432. Add the acid molecules to the simulation box, coordination numbers corresponding to the first solvation shell between HBA and HBD were increased in the binary mixture with the mole percent of CAP at 70.0%. In DES with 50% of CAP, the coordination number changes from 0.1871 to 0.3958. In contrast, DES with 50% of LUA has a coordination number of 0.2742. Decreasing CN between HBA and HBD can be attributed to the more favorable interaction of acid and salt in acid with the shorter chain length. It seems that caprylic acid molecules, which have a − 0.6269 charge on the OA atom, form a much stronger hydrogen bond with salt as compared to the LUA molecules, which have a − 0.6136 charge. RDFs between HBA and HBD were analyzed to characterize the structural correlation in aqueous solutions. In order to investigate the effect of water on the distribution of HBD around HBA, ${\mathrm{g}(\mathrm{r})}_{\mathrm{HBD }-\mathrm{ HBA}}$ was compared in pure and aqueous solutions of DES. Figure 2d shows RDFs between choline chloride salt and FAs molecules before and after mixing with water. It can be seen from Fig. 2d that the heights of peaks corresponding to ${g(r)}_{\mathrm{HBA }-\mathrm{ HBD}}$ significantly decrease in water. The decreasing trend of the CN of $g(r)$ between HBD and HBA is a confirmation of the decreasing distribution of HBD around HBA in the adjacent water (see Table S1). The coordination number related to RDF between HBA and HBD has also been used to determine structural changes in water. The structural properties of the binary mixtures may be greatly influenced by the adjacent water molecules (see Fig. 2d). The CN related to RDF between anion and HA atom of FAs for three binary mixtures containing 30, 50, and 70% of CAP is 0.1871, 0.3958, and 0.6432, respectively. The coordination number shows a significant distribution of the salt around the hydroxyl group of the FAs in the binary mixtures with a higher percentage of acid in water. Similar results have been found for the binary mixtures of LUA and [Ch^+^][Cl^−^] salt.

### Hydrogen-bond analysis

**Figure 4:**
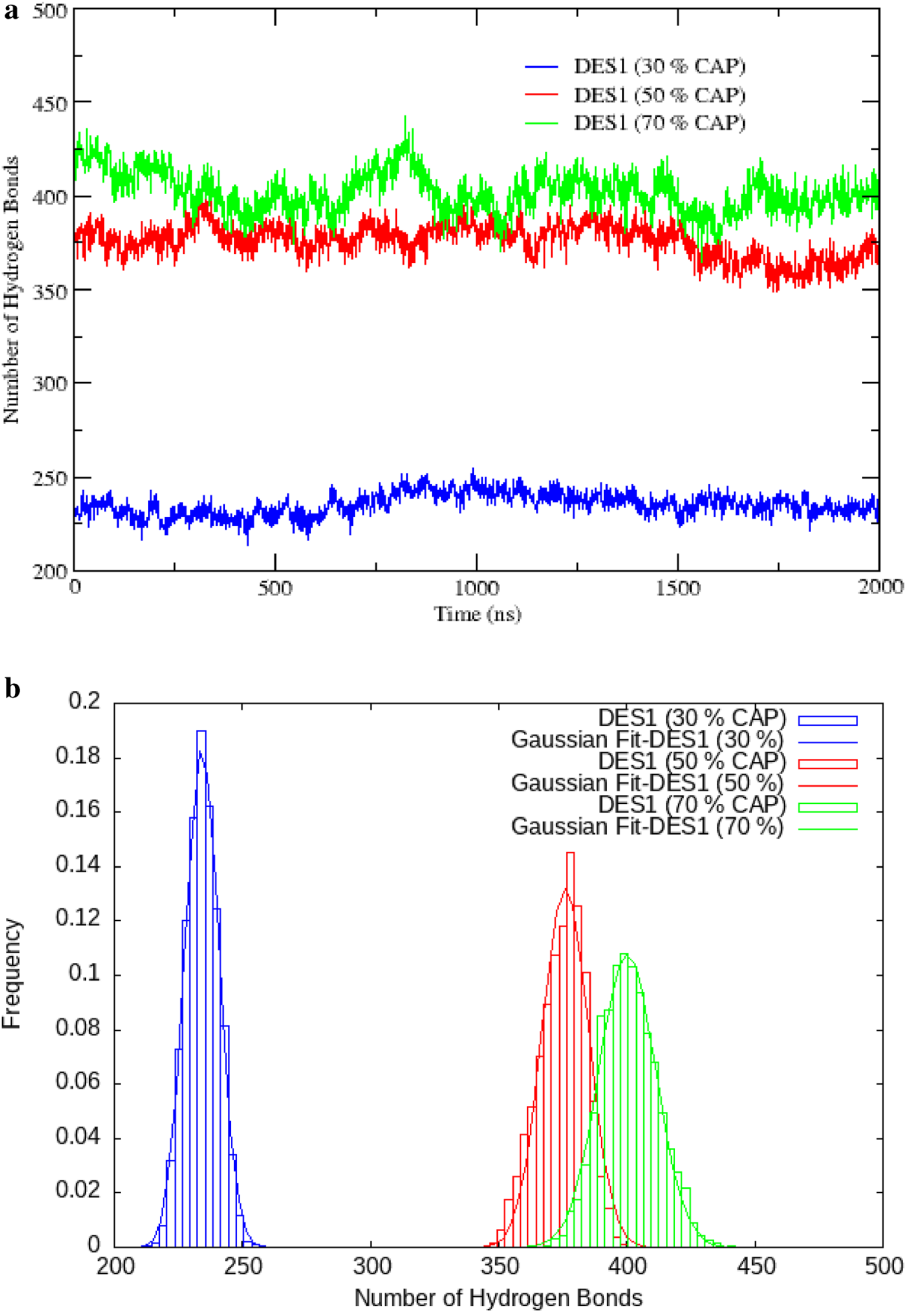
(**a**) Number of hydrogen bonds between the HA atom of CAP molecules and Cl^−^ anion at different ratios. (**b.**) The distribution of the hydrogen bond between the HA atom of CAP molecules and Cl^−^ anion at different ratios.

Hydrogen bond interactions between the HBA and HBD molecules lead to the suppression of the melting point of the eutectic solvents. The hydrogen bonding effect was calculated using the hydrogen bond plugin in VMD. Distance criteria for the formation of a hydrogen bond were determined using the position of the peak in the RDFs between species. But the distance may not be a sufficient criterion for calculating intermolecular H bonding effect. Strict criteria for the H-bond between H-bond donor molecules and H-bond acceptor molecules are determined using Combined Distribution Functions (CDF) analysis. Criterion with 2 Å/105°–120° and 2 Å/150°–180 for HA FAs–Cl^−^/HA FAs–Ch^+^ pairs are a better choice. The distribution of the hydrogen bond between the CAP molecules and choline chloride pair was drawn in the supplementary information (Fig. S2). The results show that the HA atom of the carboxyl group of FAs and Cl^−^anion has a more favorable interaction compared to choline cation. Geometric criteria for hydrogen bonding as used here, it was explained in the previous sections. In accordance with the criteria, the preferable formation of H-bonds between the (HA−O) FAs-cation and (HA–Cl^−^) FAs—anion was confirmed by the highest angular/distance probability region at 150−180°/2 Å and 105−120°/2 Å, respectively. All of the H-bond data are sorted in Table S2 (Geometrical criteria were used to find H-bonds obtained in the RDF and the CDF). Hydrogen bonds were defined by a cut-off distance of less than 3.5 Å and a cut-off angle of less than180°. For obtaining the average number of H-bonds, Gaussian functions fitted to the number of H-bond data.

3 $$\mathrm{F}\left(\mathrm{X}\right)={\frac{\mathrm{a}}{\upsigma \sqrt{2\uppi }}\mathrm{exp}}^{\frac{{-(\mathrm{X}-\overline{\mathrm{X} })}^{2}}{2{\upsigma }^{2}}}$$

where $\sigma$ is the standard deviation, $a$ and $\overline{X }$ represent the adjustable parameter and the average number of H-bond, respectively^19^. In this study, we focused on the hydrogen bonding interactions between the anion and the hydroxyl group of the cation and the -COOH group of FAs. The average number of H-bond between FAs molecules and salt is drawn in the binary mixtures with the different ratio of FAs and Ch^+^/Cl^−^ salt (see Fig. 4a,b). The average number of H-bonds between CAP molecules and chloride anions in DES with 30% of CAP was found to be 234 and that in the binary mixtures with the mole percent at 50% was 376. However, the average number of H-bond between HBA and HBD is a value of 427 at mixtures with the mole percent at 70% of acid. N_avg_ of H-bond between Lauric acid molecules and Ch^+^/Cl^−^ salt indicate strong [Ch^+^] [Cl^−^]—LUA interactions with almost 292  ± 0.0897 hydrogen bonds per acid molecule in TLA mixture with 70% of LUA. However, in the binary mixture with 70% of choline chloride is almost 197  ± 0.223 per acid molecule. We focused on the importance of hydrogen bonding interactions and the nonbonding interactions between the two components in the stability of the eutectic solvent in water. The average number of H-bonds between HBA and HBD was significant in the binary mixtures with the mole percent at 70% of FAs (CAP and LUA). However, we observed small changes in adjacent water, e.g., Number of H-bonds varied from 301to 126 for the mixture of CAP and [Ch^+^][Cl^−^] (Tabl S2).

**Figure 5:**
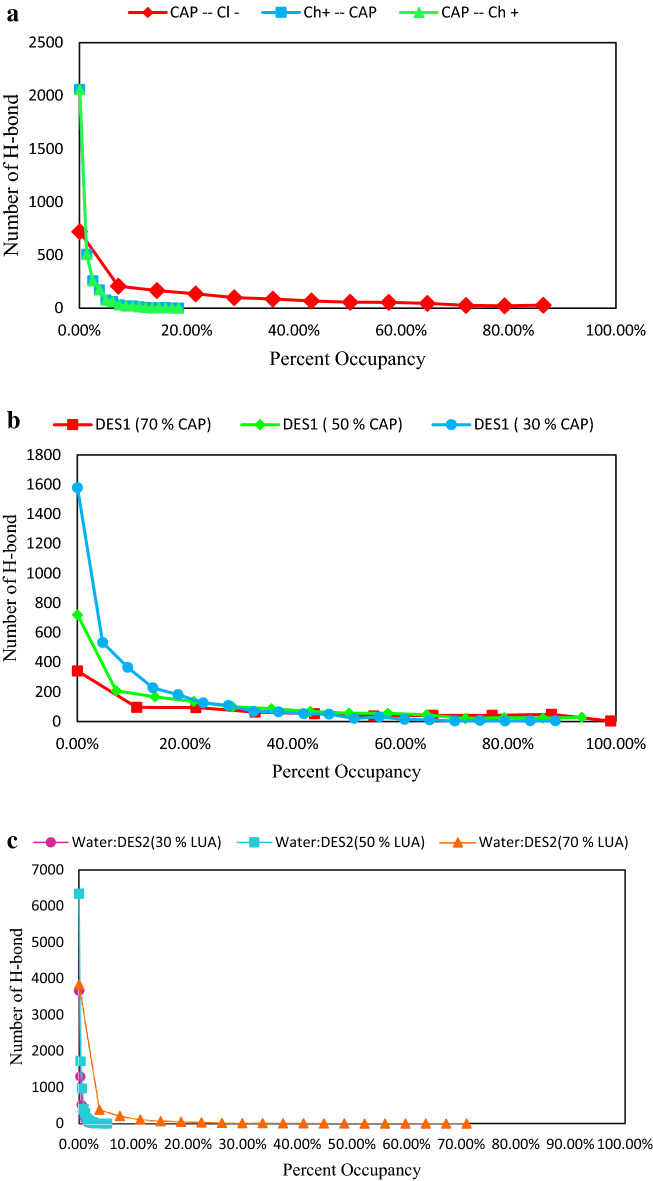
(**a**) Hydrogen bond percent occupancies for the interaction between the H atoms and Cl- anion in the mixtures containing 50% FAs at 353 K. (**b**) Hydrogen bond percent occupancies for FAs and Cl^−^ anion for the binary mixtures with the mole percent 30%, 50% and 70% FAs at 353 K. (**c**) Hydrogen bond percent occupancies for FAs and Cl^−^ anion for the binary mixtures with the mole percent 30%, 50% and 70% FAs in adjacent water.

The relative percent of occupancy was analyzed to demonstrate the total time a unique hydrogen bond between two species^20^. The persistence of hydrogen bonds are obtains by using occupancy analysis^20^. Figure 5a shows the H atom of carboxylate group, HA, of FAs has a more stable interaction with anions of HBA. The stability of hydrogen bonds between acids and choline chloride salt were investigated at mixtures with different ratio of FAs (see Fig. 5b). Choline chloride salt in mixtures with 70% acid form strong and stable hydrogen bonds with the carboxylate of FAs. Hydrogen bond interaction is disturbed for all studied systems in the adjacent water, but the persistence of hydrogen bonds of the binary mixtures with 70% of FA is high as compared to other ratios (see Fig. 5c).

### Nonbonded interaction energy and relative stability factor

Calculation of the non-bonded interaction energy is an important analysis for understanding intermolecular interactions in binary mixtures. The non-bonded energy includes terms such as short-range van der Waals (vdW) interactions and long-range electrostatic interactions which are computed Lennard–Jones (12–6) function and coulombic equation, respectively. The vdW and Coul interactions are calculated by the following equations:

4 $${E}_{Coul}=\frac{{e}^{2}}{4\pi {\varepsilon }_{0}}\sum_{i\ne j}\frac{{q}_{i}{q}_{j}}{{r}_{ij}}$$

5 $${E}_{vdW}={\sum }_{i\ne j}{D}_{0,ij}\left[{\left(\frac{{R}_{0,ij}}{{r}_{ij}}\right)}^{12}-2{\left(\frac{{R}_{0,ij}}{{r}_{ij}}\right)}^{6}\right]$$

where ${q}_{i} and {q}_{j}$ represent the atomic partial charges. D_o_ and R_o_ are the depth of the potential and the equilibrium atomic separation, respectively^21^.

The nonbonded interaction energies between different species of each system are listed in Table 2. In this section, a relative measure was computed to quantify the stability of DESs based on choline chloride and fatty acid in an aqueous solutions. To obtain the relative stability of hydrophobic DESs in water, the interaction energies between HBA–HBD, HBA-water, and HBD-water pairs were discussed in different ratios of fatty acids (caprylic acid and Lauric acid) and choline chloride. Relative stability is related to the stability of DES systems in water. Since the interaction energies between water and thymol are almost equal, it follows that the stability factor was a function of the HBD−water interaction . The relative stability factor (S) is defined as:

**Table 2:** MD Simulated Non-bonded Interaction Energies (Van der Waals, Evdw, Electrostatic, Ecoul, and intermolecular, Einter, kcal/mol) between the Different Pairs of HBA−HBD−Water for the Different Systems Calculated at 353 K.

n HBA: n HBD	Component pairs	CCA			Relative stability factor (S)
		E_vdw_	E_Coul_	E_total_	
300: 700	Salt–water	− 6.04	− 12.5565	− 18.5965	0.107255
	Salt–FAs	− 13.1565	− 23.53296	− 29.5729	
	FAs–water	− 7.66	− 249.4691	− 257.1292	
500: 500	Salt–water	− 5.3200	− 139.9626	− 145.2826	0.327813
	Salt–FAs	− 7.66	− 116.40756	− 124.0676	
	FAs–water	− 10.2	− 222.9875	− 233.1876	
700: 300	Salt–water	− 2.5	− 116.04365	− 118.5436	0.878032
	Salt–FAs	− 7.34	− 173.7855	− 181.1255	
	FAs–water	− 16.26	− 79.8365	− 96.0965	

n HBA: n HBD	Component pairs	CLA			Relative stability factor (S)
		E_vdw_	E_Coul_	E_total_	
300: 700	Salt–water	− 20.42	− 94.5893	− 115.0094	0.36219
	Salt–FAs	0.3612	− 137.4788	− 137.1176	
	FAs–water	− 7.84	− 255.7198	− 263.55988	
500: 500	Salt–water	− 5.03	− 174.7148	− 179.7449	0.680039
	Salt–FAs	1.06	− 196.7638	− 195.70383	
	FAs–water	− 14.93	− 93.1084	− 108.0385	
700: 300	Salt–water	− 3.6930	− 193.606	− 197.2936	0.843856
	Salt–FAs	− 5.23	− 304.53	− 309.7675	
	FAs–water	− 16.77	− 127.1285	− 143.8986	

6 $$S=\frac{IE(HBA-HBD)}{IE \left(HBA-water\right)+IE(HBD-water)}$$

To study the non-bonded interaction energy, Cl^−^−Water. H, Ch^+^−Water. H, and FAs. HA−Water. O interactions were selected due to the distributions of water molecules around carboxylate (–COO–) of FAs, hydroxyl (OH–) of Ch^+^ cations, and chloride anion. E _vdW_ and E _Coul_ amounted to − 7.23 kcal/mol and − 116.044 kcal/mol for HA. Fas–Cl^−^ interaction in the binary mixture with 70% of FAs at the adjacent water, which E_total_ is 31% more than the interaction energy of anions and CAP molecules in the binary mixture with 50% of FAs. To test the possibility that binary mixtures with a higher percentage of FAs composition are very stable in aqueous solutions, E_total_ between HBA and HBD was compared for the binary mixtures with a molar percentage of 30%, 50 and 70% of FAs (ie. luaric acid and caprylic acid) at the adjacent of water. The results showed that the interaction between FAs molecules and Cl^−^ anion was much more favorable in the binary mixture containing 70% of FAs than in other percentages.

It should be noted that, the value of the E _total_ between LUA molecules and anion is than that of the LUA. H–Ch^+^.O interaction energy in the binary mixture at pure state. Furthermore, the E _Coul_ contribution decreased an order of magnitude lower in LUA. H–Cl^−^ interaction. In general, It seems that the choline chloride salt has a higher affinity toward the water-rich phase compared to fatty acids, so the interaction between the FAs molecules and the Cl^−^ anion is dramatically reduced in the presence of water. However, the stability factor is a more suitable parameter to investigate the stability of binary mixtures in water. The stability factor values were calculated by using MD simulation (see Table 2). The stability factor values calculated on the basis of the simulated result is in range between 0.1 and 0.9. According to these results, the binary mixtures with a molar percentage of 70% of FAs have more stability in the adjacent water.

### Spatial distribution functions

**Figure 6:**
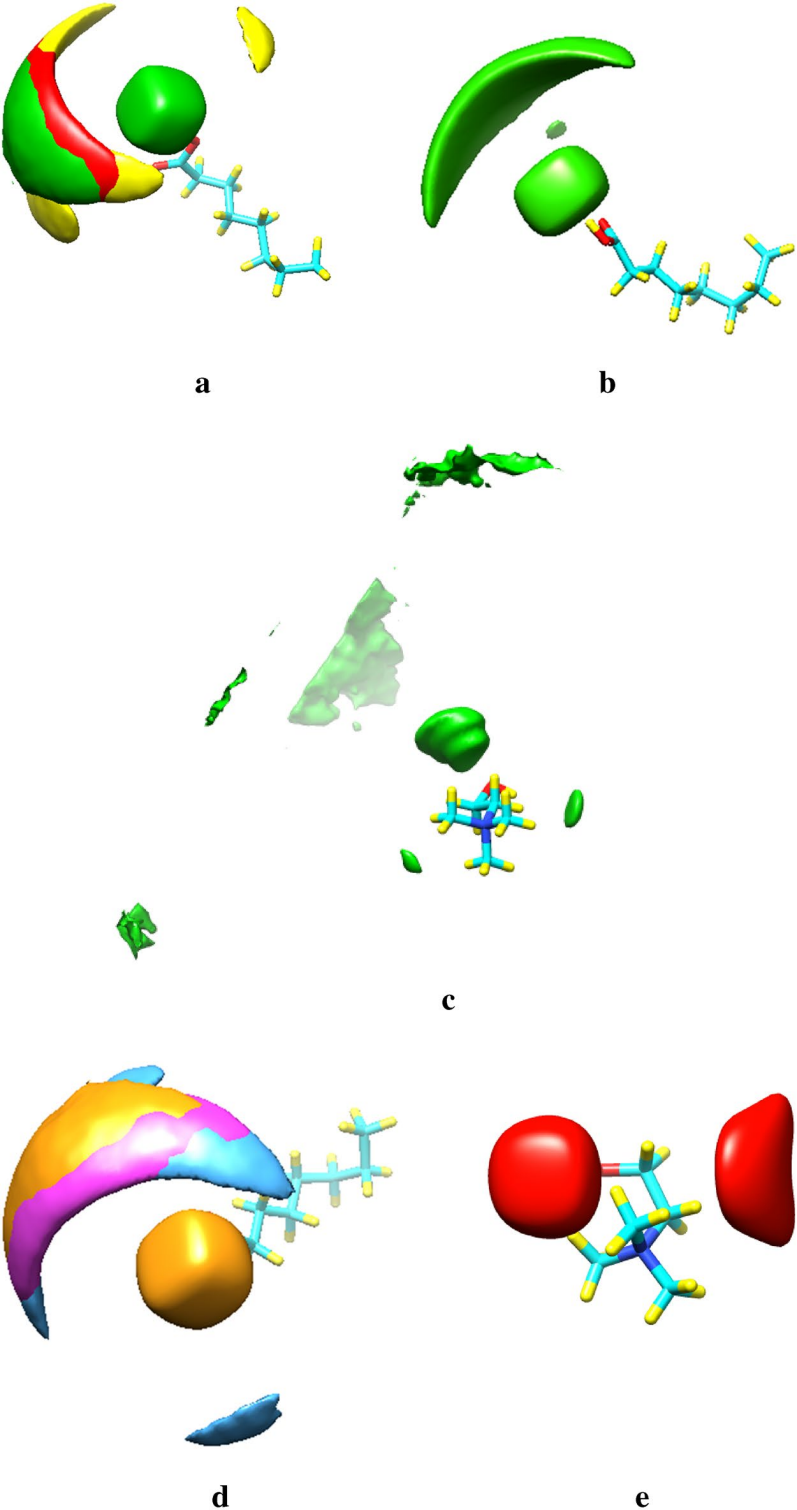
Spatial distribution functions (SDFs) of the eutectic mixture components with 70% FAs at 353 K. yellow isosurfaces correspond with choline cations, green isosurfaces are chloride anions, and red isosurfaces are FAs molecules.

The spatial distribution function (SDF) of the species around each other was obtained through the TRAVIS package. The SDF analysis was used for understanding the microscopic structure of the binary mixtures in the adjacent water^22^. Green isosurfaces correspond with the spatial distribution of the anion around the FAs molecule, yellow isosurfaces are the distribution of the choline cation around reference FAs and red isosurfaces are the spatial distribution of fatty acid molecules around significant FAs in the binary mixtures with the mole percent at 50% of CAP (see Fig. 6(Panel a)). As can be appreciated from the colored SDFs for different species, the more active (–COOH) site of FAs molecules is surrounded by Cl^−^ anions in the binary mixtures (see Fig. 6(Panel b)). The highly distributed anion around the FAs molecule can be seen in Fig. 6(Panel c). It should be noted that the anions are mainly located on the inner surface of the distribution of species around reference FAs molecules. Also, the distribution of acid molecules around choline is remarkable in the binary mixture.There are almost no significant changes in the distribution process of species with changing the number of acids from 300 to 500 in the box simulation. However, an isosurface shows that there is a rather high degree of association of HBA and HBD in the TLA with 70% LUA. Such a trend might appear reasonable since the spatial distribution of the anion around the cation was decreased in this ratio (see Fig. 6(Panel d)). SDFs reveal the fact that the distribution of species around each other is disturbed in water. The distribution of HBA around the central HBD gradually decreases as the binary mixture becomes more dilute (see Fig. 6(Panel c)). It seems that Cl^−^ anions and Ch^+^ cations prefer to be positioned near the H atoms of water molecules in the water/DES mixtures. However, by adding water molecules to the simulation box, the correlations between HBA and HBD were weak in the binary mixtures of 30% of CAP and 30% of LUA (see Fig. 6(Panel c)). The water molecules are evenly distributed around salt. However, It seems the distribution of water molecules around the FAs to be negligible in the binary mixtures with a molar percentage of 70% FAs (see Fig. 6(Panel d)).

### Dynamic and transport properties

#### Rotational and lateral diffusion, mean square displacement

Accurate predictions of the dynamic behavior and the microscopic motion of the species will be interesting topics for future studies due to the widespread use of DESs in industry^23^. The dynamic properties of the eutectic solvents were also estimated from the self-diffusion coefficients of species by using the Einstein relation. Self-diffusion coefficients, D_self_, given by:

7 $${D}_{\mathrm{self}}=\underset{\uptau \to \infty }{\mathrm{lim}}\frac{1}{6\uptau }{\langle {\left|{\overrightarrow{\mathrm{r}}}_{\mathrm{i}}\left(\mathrm{t}+\uptau \right)-{\overrightarrow{\mathrm{r}}}_{\mathrm{i}}(\mathrm{t})\right|}^{2}\rangle }_{\mathrm{t},\mathrm{i}}$$

where $\langle {\left|{\overrightarrow{\mathrm{r}}}_{\mathrm{i}}\left(\mathrm{t}+\uptau \right)-{\overrightarrow{\mathrm{r}}}_{\mathrm{i}}(\mathrm{t})\right|}^{2}\rangle$ stands for the mean-square displacement (MSD) of species *i*. The MSD is defined as

8 $$\mathrm{MSD}\left(\uptau \right)={\langle {\left|{\overrightarrow{\mathrm{r}}}_{\mathrm{i}}\left(\mathrm{t}+\uptau \right)-{\overrightarrow{\mathrm{r}}}_{\mathrm{i}}(\mathrm{t})\right|}^{2}\rangle }_{\mathrm{t},\mathrm{i}}$$

The well-known $\beta$ exponent was used to determine the location of the diffusive regime by Del Pópolo and Voth^24^. To determine the $\beta$ parameter, the slope of MSD versus simulation time, was calculated as

9 $$\upbeta =\frac{{\mathrm{dlog}}_{10}<\mathrm{\Delta r}({\mathrm{t})}^{2}>}{{\mathrm{dlog}}_{10}\mathrm{t}}$$

**Figure 7:**
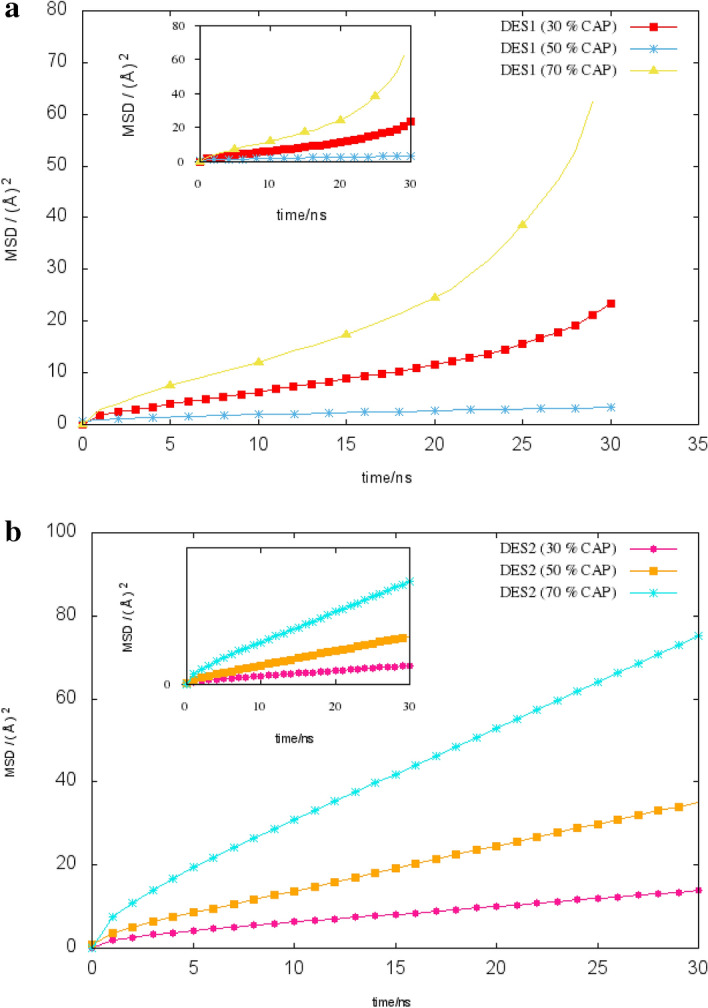
(**a**) The MSDs of chloride anions for the mixtures containing 30%, 50% and70% of CAP at 353 K. (**b**) The center of mass MSDs of FAs of the mixture containing 50% of LUA at 353 K.

According to the opinions of Del Pópolo and Voth^25^, ${D}_{\mathrm{self}}$ was calculated from the MD simulations at the diffusive regime $\left(\beta =1\right)$. Self-diffusion coefficients (D_self_) of species were calculated from the slopes of MSD-time curves. The D_self_ data are listed in Table 3. D_self_ of Cl^−^ anion is 0.2214, 0.4172 and 0.1927 ${{\mathrm{A}}^{^\circ }}^{2} {\mathrm{ns}}^{-1}$ or the binary mixtures with molar percentage of 30%, 50% and 70% CAP, respectively. D_self_ of Cl^−^ anion is 0.1927 ${{\mathbf{A}}^{^\circ }}^{2}{\mathbf{n}\mathbf{s}}^{-1}$ in the binary mixtures with 70% of CAP, while the D_self_ of Cl^−^ corresponds to the same ratio in adjacent water is 124.2521 ${{\mathbf{A}}^{^\circ }}^{2}{\mathbf{n}\mathbf{s}}^{-1}$. When comparing the D_self_ of species, we observed significant changes in the D_self_. The mean-square displacement (MSD) analysis is a very useful for tracking atomic motion. The mean square displacements (MSD) of species were plotted in the binary system versus the simulation time at 0−30 ns. The MSD functions at the binary mixture with a molar percentage of 70% LUA exhibit a hydrogen-bonding network that strongly limits the migration of molecules. There is one strong inermolecular O–H⋯Cl^−^ hydrogen bond (FAs.H …Cl^−^), resulting in a decrease in the slope of MSDs. These results are very good qualitative agreement with results of Combined Distribution Functions analysis (Fig. 7a,b).

**Table 3:** The calculated self-diffusion coefficient of the species from the slope of MSD plots for the binary mixtures.

n [Ch^+^ [Cl^−^]: n FAs	D Å^2^ ns^−1^		
	CCA		
	Ch^+^	Cl^−^	FAs
300 700	0.0754	0.2214	0.27035
500 500	0.3968	0.4172	2.3433
700 300	0.01581	0.0144	0.0198
	TLA		
300 700	0.0951	0.0554	0.2434
500 500	0.2147	0.1934	0.6476
700 300	0.192	0.1920	0.5825

### Thermo-physical properties analysis

To obtain the shear viscosity in molecular dynamics simulation, the shear autocorrelation function was calculated using the Green–Kubo method^26^. The shear viscosity is given by the following expression:

10 $$\upeta =\frac{\mathrm{V}}{{k}_{B}T}{\int }_{0}^{\infty }dt<{P}_{xy}\left(0\right){P}_{xy}\left(t\right)>,$$

where η is the shear viscosity, V and T represent the volume and the temperature of system, respectively. ${\mathrm{k}}_{\mathrm{B}}$ is the Boltzmann constant.${P}_{xy}$ refers the off-diagonal element of the stress tensor^27^. After 50 ns NPT, five short runs, each of 1 ns in length, was performed with storing each frame. The shear viscosities for the studied systems are presented in Table 4. The shear viscosity of the binary mixture with 30% CAP at 353 K is 6.398 $\mathrm{mPa S}$ while adding FAs molecules increased the viscosity of the binary mixture at 70% CAP (see Table 4). Similarly, a small change in the shear viscosity was observed by changing the percentage of LUA from 30 to 50%. So, the binary mixtures with a molar percentage of 70% have the greatest shear viscosity. There is evidence of strong hydrogen bonding between Cl^−^ anions of HBA and the headgroup of HBD at the binary mixtures with 70% FAs.

**Table 4:** The density distribution function (dens) and the shear viscosity (η) of the binary mixtures from simulations.

n [Ch^+^ Cl^−^]: n FAs	Binary mixtures of CAP and [Ch^+^][Cl^−^]		
	Dens /distance (Å)		η/mPa s
	R	Dens	
300 700	2.10	0.18690615	6.398
500 500	2.10	0.558633128	16.444
700 300	2.10	0.937019495	26.309

### Velocity autocorrelation functions

The dynamics behavior of species in the binary mixtures can be studied by the normalized velocity autocorrelation function (VACF).${C}_{v}$ can be calculated from the follows equation:

11 $${C}_{v}\left(t\right)=\frac{\langle {V}^{c}(t).{V}^{c}(0)\rangle }{\langle {V}^{c}(0).{V}^{c}(0)\rangle }$$

where ${V}^{c}\left(t\right)$ related to the center of mass velocity of specie and the angular brackets $\langle \rangle$ is ensemble average over all time origins^28^.

**Figure 8:**
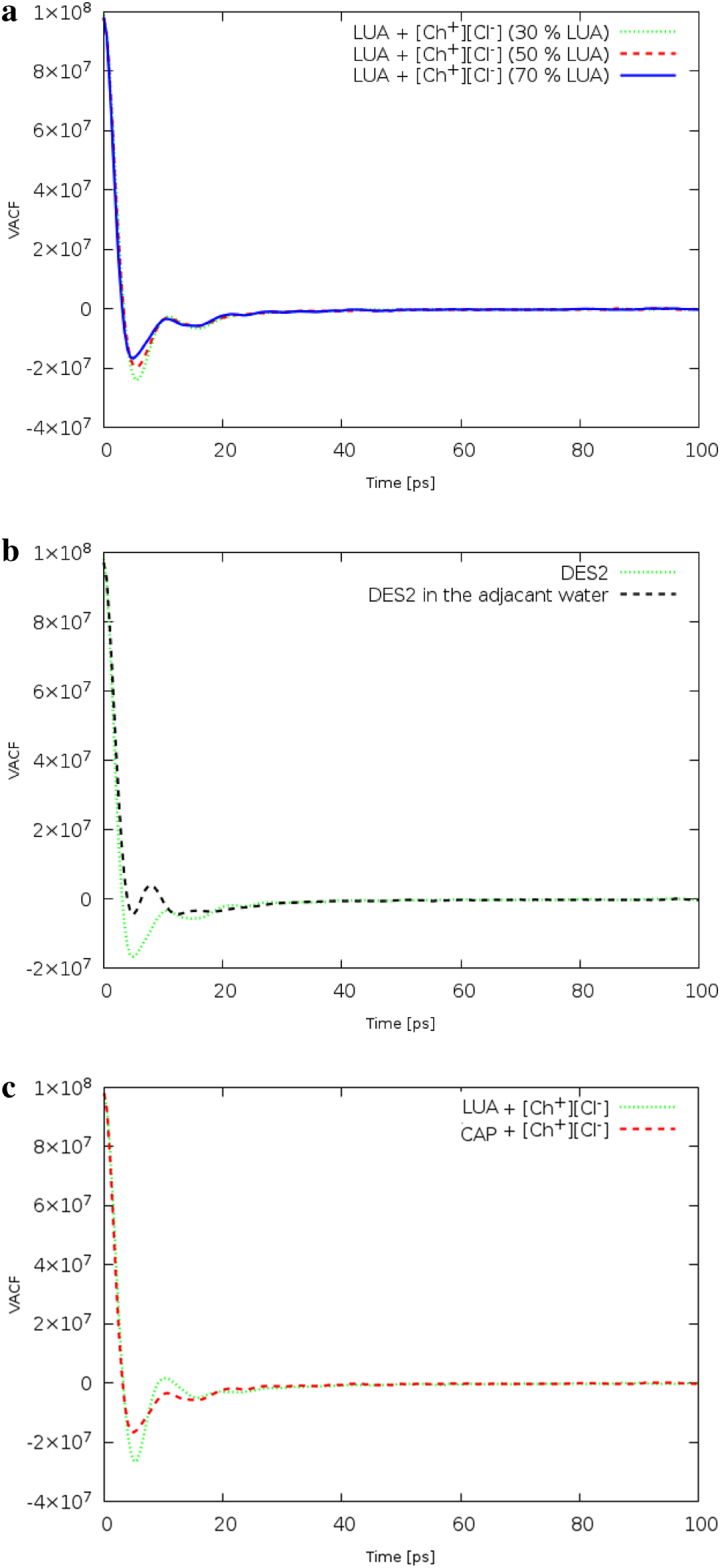
(**a**) The dependence of the VACFs of chloride anions on the solvent percent composition at 353 K. (**b**) The VACFs of chloride anions for the pure [Ch^+^/Cl^−^][FAs] DES and the binary mixtures containing 50% in adjacent water. (**c**) The VACFs of chloride anions for the [Ch^+^/Cl^−^][FAs] mixtures containing 70% FAs.

A total of five simulation runs were performed for each of 1 ns in length. The input files of short runs were prepared from the latest step 50 ns in the ensemble NPT. To calculate VACFs, velocity data were collected in three directions in the x-axis, y-axis, and z-axis directions, with an interval of 10 ns. In order to confirm the statistical accuracy of the VACFs results and improve their reproducibility, an average of five runs was conducted for each of the studied systems. The computed VACFs of species for the binary mixture of FAs and [Ch^+^] [Cl^−^] with the molar percentage of 50% CAP are compared in Fig. S5. VACFs of species presented here show that Cl^−^ anions velocities randomizes sooner than cation and FAs molecules. The point here is that the second zero is the velocity randomization time in the VACFs. The mean collision times (first zero) for Cl^−^ anions at the molar percentage of 70% CAP are estimated at around 5 ps, whereas, the value of this quantity for a similar molar percentage is around 50 in the binary mixture of LUA and [Ch^+^] [Cl^−^] (Fig. 8a).The depth well of the first minimum of VACF for the binary mixtures with the molar percentage of 70% CAP confirms the high density in this ratio (see Table 4). The first depth well of VACF was decreased by adding water molecules to the simulation box. So that the negative region of the VACF cannot be clearly distinguished in water (Fig. 8b,c).

### Vector reorientation dynamics

Vector reorientation dynamics were calculated for any vector O–H corresponding to the molecules. VRD(τ) provides some information about the fast of vector orientation of species in the system. VRD(τ) is computed using Eq. (12):

12 $$VRD\left(\tau \right)=N.{\left\langle \sum_{t=0}^{T-\tau }{\overrightarrow{a}}_{i}\left(t\right).{\overrightarrow{a}}_{i}(t+\tau )\right\rangle }_{i }$$

**Figure 9:**
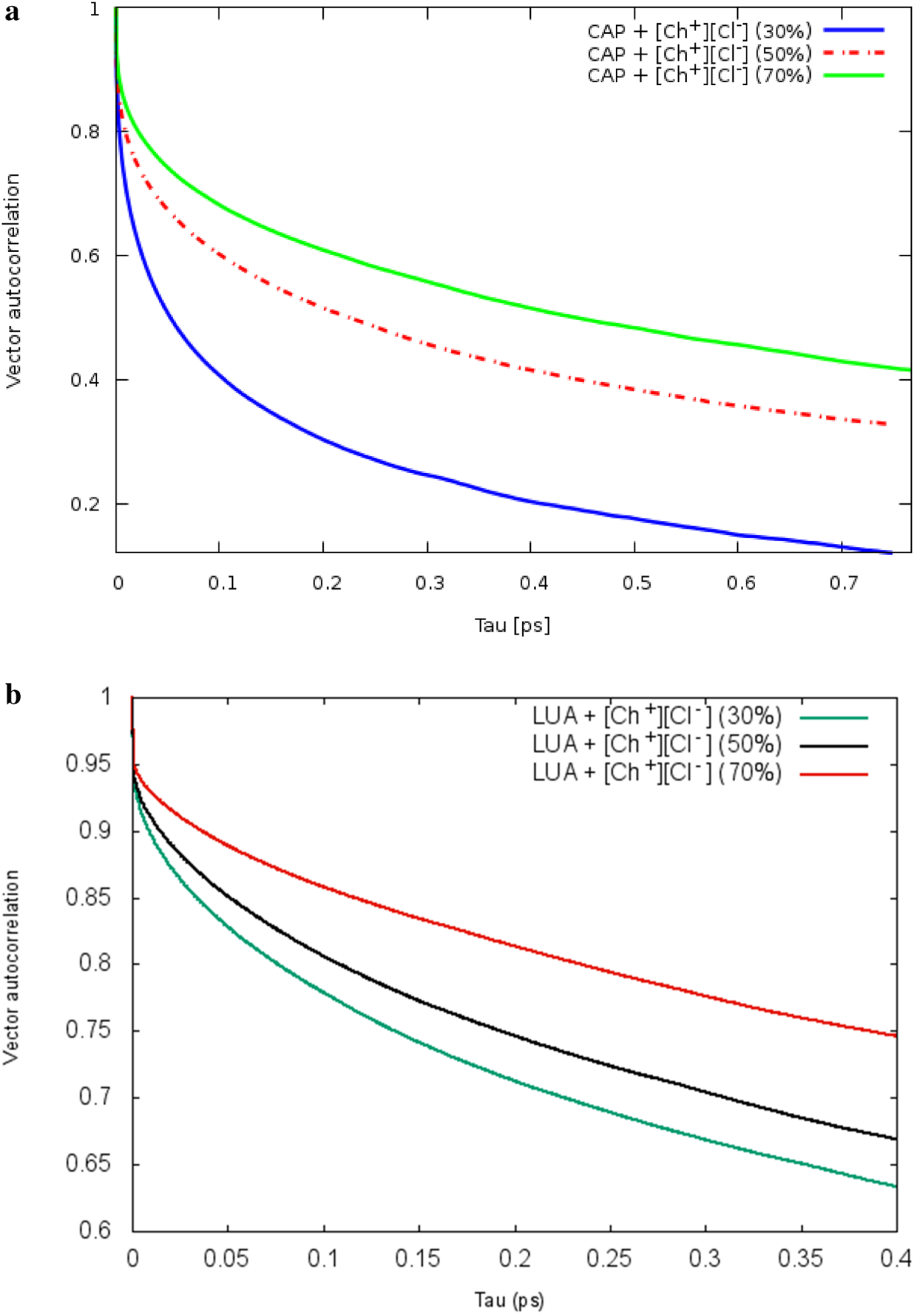
(**a**) Vector reorientation dynamics for bond (O1–HA) for mixtures at the different ratios of CAP:Ch^+^ Cl^−^. (**b**) Vector reorientation dynamics for bond (O1–HA) for mixtures at the different ratios of LUA:Ch^+^ Cl^−^.

**Figure 10:**
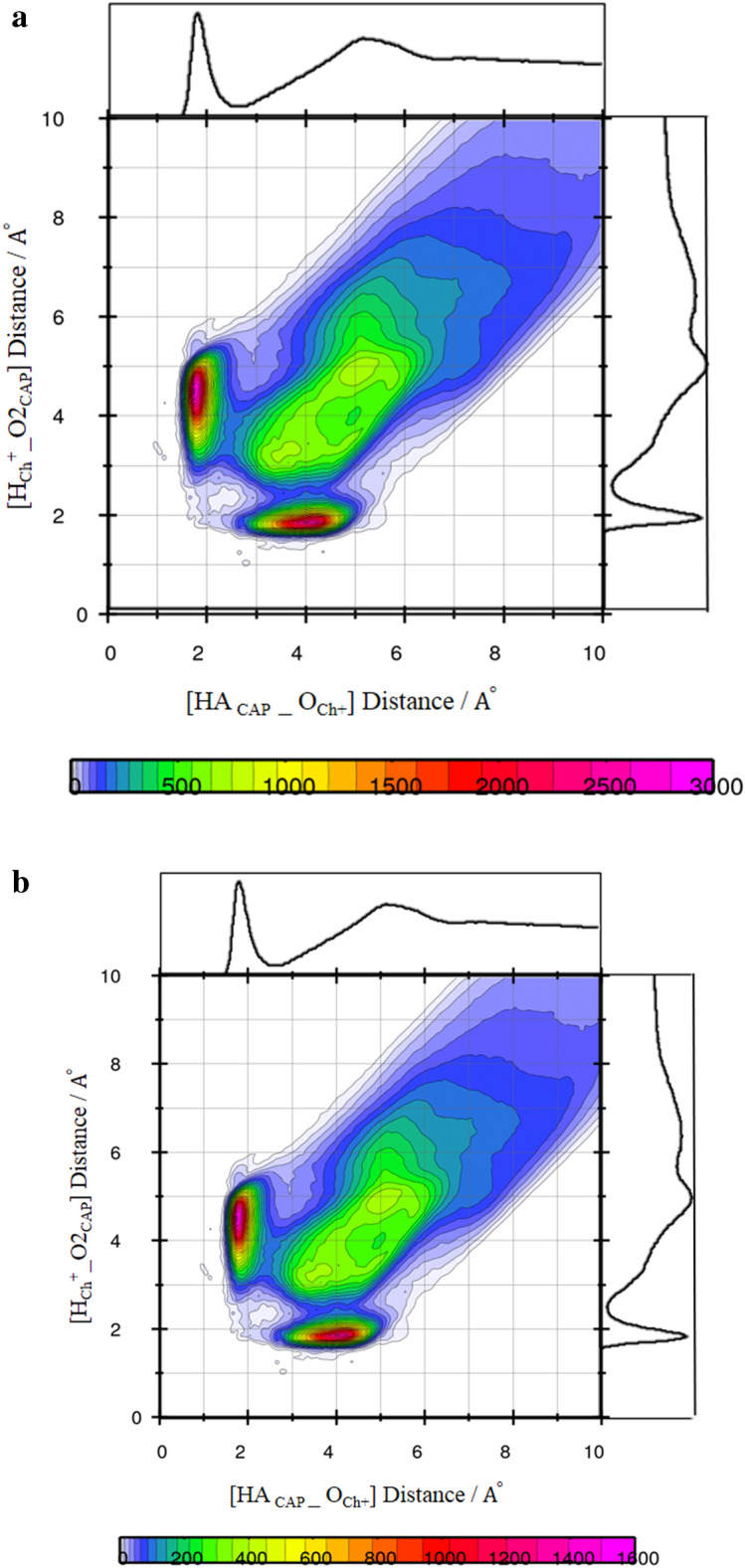
(**a**) Combined radial/radial distribution function between Ch^+^ and CAP molecules in the binary mixtures at pure state. (**b**) Combined radial/radial distribution function between Ch^+^ and CAP molecules in the binary mixtures at the adjacent water.

The VRD(τ) is often defined as the normalized sum over the dot product between the vector at some time t, ${\overrightarrow{a}}_{i}\left(t\right)$, and the same vector at some later time t + τ, ${\overrightarrow{a}}_{i}\left(t+\tau \right)$ for all starting times t^29^. To examine the reorientation of vectors, we have chosen the following bond vector. In the binary mixtures, the reorientation dynamics of the different vector of CAP molecules are visualized in Fig. 9a. The blue, red, and green lines show the reorientation of OA–HA bond vectors within each of the FAs molecules at the binary mixtures with the binary mixtures with 30, 50 and 70% of CAP , respectively. It can be seen that the orientation of OA — HA bond vectors in the binary mixtures with 30 and 50% of FAs is faster than that of the binary mixtures with 70% of FAs (see Fig. 9b and Fig. S5). The first peak of the cation—FAs and anion—FAs RDFs in the binary mixtures with 70% of FAs is estimated at around 2.11 and 2 Å, respectively. The hydroxyl group of FAs has a significant role in the structural correlations of the binary mixtures. The intermolecular H-bonding interactions (O–H···O or O–H···Cl^−^) can lead to vector reorientation. The formation of H-bonding between water and choline cation leads to decreases in the occurrence probability of the [Ch]^+^···[FAs] ···[Ch]^+^ structure in water (see Fig. 10a,b). It seems that the orientation of bond vectors is influenced by water molecules.

## Conclusion

MD simulation was used to investigate the stability of DESs based on fatty acids and choline chloride in the adjacent water. For this purpose, the structural and dynamic properties of binary mixtures were investigated at different ratio of FAs and [Ch^+^][Cl^−^]. From the study on the structural properties of solvents, we infer that chloride anions play a key role in the formation of DESs. We mention that the structural properties were indicated in the mixtures with 70% of FAs interaction between HBA and HBD compared to the different percentages of FAs. It seems that chloride anion plays a key role in the formation of eutectic solvents based on [Ch^+^] [Cl^−^] and FAs. Furthermore, a preferred arrangement of [Ch]^+^ cations around FAs molecules is due to the stability of the H-bond between the hydroxyl oxygen of cations and hydrogen atoms of carboxyl (–COOH) of FAs. Significant differences in intermolecular interaction of DESs were observed in the adjacent water. However, the binary mixtures containing 70% FAs, which anions have tend to form strong hydrogen bonds, are commonly the very stable mixture in water.

## Supplementary Information

Supplementary Information.

## Data Availability

All data generated or analyzed during this study are included in this published article.

## References

[1] Zhang L, Wang M (2017). Optimization of deep eutectic solvent-based ultrasound-assisted extraction of polysaccharides from *Dioscorea opposita* Thunb. Int. J. Biol. Macromol..

[2] Makoś P, Przyjazny A, Boczkaj G (2018). Hydrophobic deep eutectic solvents as “green” extraction media for polycyclic aromatic hydrocarbons in aqueous samples. J. Chromatogr. A.

[3] Martins MA, Pinho SP, Coutinho JA (2019). Insights into the nature of eutectic and deep eutectic mixtures. J. Solution Chem..

[4] Ribeiro BD, Florindo C, Iff LC, Coelho MA, Marrucho IM (2015). Menthol-based eutectic mixtures: Hydrophobic low viscosity solvents. ACS Sustain. Chem. Eng..

[5] Buzzeo MC, Evans RG, Compton RG (2004). Non-haloaluminate room-temperature ionic liquids in electrochemistry—–A review. J. ChemPhysChem..

[6] Zhang Z, Liu W, Xie H, Zhao ZK (2011). An unexpected reaction between 5-hydroxymethylfurfural and imidazolium-based ionic liquids at high temperatures. Molecules.

[7] Abbott AP, Capper G, Davies DL, Rasheed RK, Tambyrajah V (2003). Novel solvent properties of choline chloride/urea mixtures. Chem. Commun..

[8] Dwamena AK (2019). Recent advances in hydrophobic deep eutectic solvents for extraction. Separations..

[9] Florindo C, Branco L, Marrucho I (2017). Development of hydrophobic deep eutectic solvents for extraction of pesticides from aqueous environments. Fluid Phase Equilib..

[10] Frisch, M.J., Trucks, G., Schlegel, H., Scuseria, G., Robb, M., Cheeseman, J., Scalmani, G., Barone, V., Mennucci, B., & Petersson, G. Gaussian 09, Revis. B. 01, Gaussian. Inc., Wallingford *CT* 2009, 1–20. (https://gaussian-09w.software.informer.com/).

[11] Barani Pour S, Sardroodi JJ, Ebrahimzadeh AR (2022). Structure and dynamics of thymol-fatty acids based deep eutectic solvent investigated by molecular dynamics simulation. Fluid Phase Equilib..

[12] Martínez L, Andrade R, Birgin EG, Martínez JM (2009). PACKMOL: A package for building initial configurations for molecular dynamics simulations. J. Comput. Chem..

[13] Phillips JC, Braun R, Wang W, Gumbart J, Tajkhorshid E, Villa E (2005). Scalable molecular dynamics with NAMD. J. Comput. Chem..

[14] Shi B, Sinha S, Dhir VK (2006). Molecular dynamics simulation of the density and surface tension of water by particle-particle particle-mesh method. J. Chem. Phys..

[15] Mendez-Morales T, Carrete J, Bouzon-Capelo S, Perez-Rodriguez M, Cabeza O, Gallego LJ (2013). MD simulations of the formation of stable clusters in mixtures of alkaline salts and imidazolium-based ionic liquids. J. Phys. Chem. B.

[16] Brehm, M., & Kirchner, B. TRAVIS-a free analyzer and visualizer for Monte Carlo and molecular dynamics trajectories. ACS Publications (2011) (http://www.travis-analyzer.de/). 10.1021/ci200217w 21761915

[17] Kroon J, Kanters J (1974). Non-linearity of hydrogen bonds in molecular crystals. Nature.

[18] Katre G, Joshi C, Khot M, Zinjarde S, RaviKumar A (2012). Evaluation of single cell oil (SCO) from a tropical marine yeast Yarrowia lipolytica NCIM 3589 as a potential feedstock for biodiesel. AMB Express.

[19] Barani Pour S, Sardroodi JJ, Ebrahimzadeh AR (2022). Structure and dynamics of hydrophobic deep eutectic solvents composed from terpene-fatty acids investigated by molecular dynamics simulation. J. Mol. Graph. Modell..

[20] Perkins SL, Painter P, Colina CM (2014). Experimental and computational studies of choline chloride-based deep eutectic solvents. J. Chem. Eng. Data.

[21] Benazzouz B, Zaoui A (2012). Thermal behaviour and superheating temperature of Kaolinite from molecular dynamics. Appl. Clay Sci..

[22] Dočkal J, Svoboda M, Lísal M, Moučka F (2019). A general hydrogen bonding definition based on three-dimensional spatial distribution functions and its extension to quantitative structural analysis of solutions and general intermolecular bonds. J. Mol. Liq..

[23] Jahanbin Sardroodi J, Rastkar Ebrahimzadeh A, Avestan MS (2022). Structural and dynamic properties of eutectic mixtures based on menthol and fatty acids derived from coconut oil: A MD simulation study. Sci. Rep..

[24] Atilhan M, Aparicio S (2017). Behavior of deep eutectic solvents under external electric fields: A molecular dynamics approach. J. Phys. Chem. B.

[25] Del Pópolo MG, Voth GA (2004). On the structure and dynamics of ionic liquids. J. Phys. Chem. B.

[26] Barani Pour S, Jahanbin Sardroodi J, Rastkar Ebrahimzadeh A, Sadegh Avestan M (2022). Using molecular dynamics simulations to understand the effect of fatty acids chain length on structural and dynamic properties of deep eutectic solvents based on choline chloride and fatty acids. ChemistrySelect.

[27] Nevins D, Spera F (2007). Accurate computation of shear viscosity from equilibrium molecular dynamics simulations. Mol. Simul..

[28] Kowsari MH, Tohidifar L (2016). Tracing dynamics, self-diffusion, and nanoscale structural heterogeneity of pure and binary mixtures of ionic liquid 1-hexyl-2, 3-dimethylimidazolium bis (fluorosulfonyl) imide with acetonitrile: insights from molecular dynamics simulations. J. Phys. Chem. B.

[29] Barani Pour S, Sardroodi JJ, Ebrahimzadeh AR (2021). The study of structure and interactions of glucose-based natural deep eutectic solvents by molecular dynamics simulation. J. Mol. Liq..

