# Supplementary material for: Investigation the effect of water addition on intermolecular interactions of fatty acids-based deep eutectic solvents by molecular dynamics simulations
Source: Sci Rep. 2023 May 8;13:7433. doi: 10.1038/s41598-023-33234-8 (PMC10167259; doi:10.1038/s41598-023-33234-8)
Supplement: Supplementary file 1 — Supplementary Information. [file 41598_2023_33234_MOESM1_ESM.docx]

Table S1. Coordination Numbers of HBD around HBA in pure [Ch^+^/Cl^-^][FAs] DES and the binary mixtures in adjacent water.

|  | HBA----HBD |  | HBA----HBD |  |
| --- | --- | --- | --- | --- |
|  | TCA |  | TCA in adjacency water |  |
| n [Ch^+^ Cl] : n FAs | r(Å) | N_coor_ | r(Å) | N_coor_ |
| 300 : 700 | 2.11 | 0.1871 | 2.18 | 0.0141 |
| 500 : 500 | 2.11 | 0.3958 | 2.18 | 0.011 |
| 700 : 300 | 2.11 | 0.6432 | 2.18 | 0.0604 |
|  | TLA |  | TLA in adjacency water |  |
| 300 : 700 | 2.15 | 0.1221 | 2.18 | 0.0141 |
| 500 : 500 | 2.15 | 0.2742 | 2.18 | 0.0363 |
| 700 : 300 | 2.15 | 0.6205 | 2.18 | 0.2182 |

**Table S2.** The average number of hydrogen bonds between the species (Navg) in pure [Ch^+^/Cl^-^][FAs] DES and the binary mixtures in adjacent water.

| n [Ch+ Cl] : n FAs | TCA | TCA in adjacency water |
| --- | --- | --- |
|  | HBA – HBD | HBA – HBD |
| 300 : 700 | 234 +/- 0.136 | 15 +/- 0.1431 |
| 500 : 500 | 376 +/- 0.3887 | 269 +/- 0.2489 |
| 700 : 300 | 427 +/- 0.2321 | 301 +/- 0.2487 |
|  | TLA | TLA in adjacency water |
| 300 : 700 | 197 +/- 0.223 | 21 +/- 0.0801 |
| 500 : 500 | 230 +/- 0.495 | 41 +/- 0.1208 |
| 700 : 300 | 292 +/- 0.0897 | 126 +/- 1.287 |


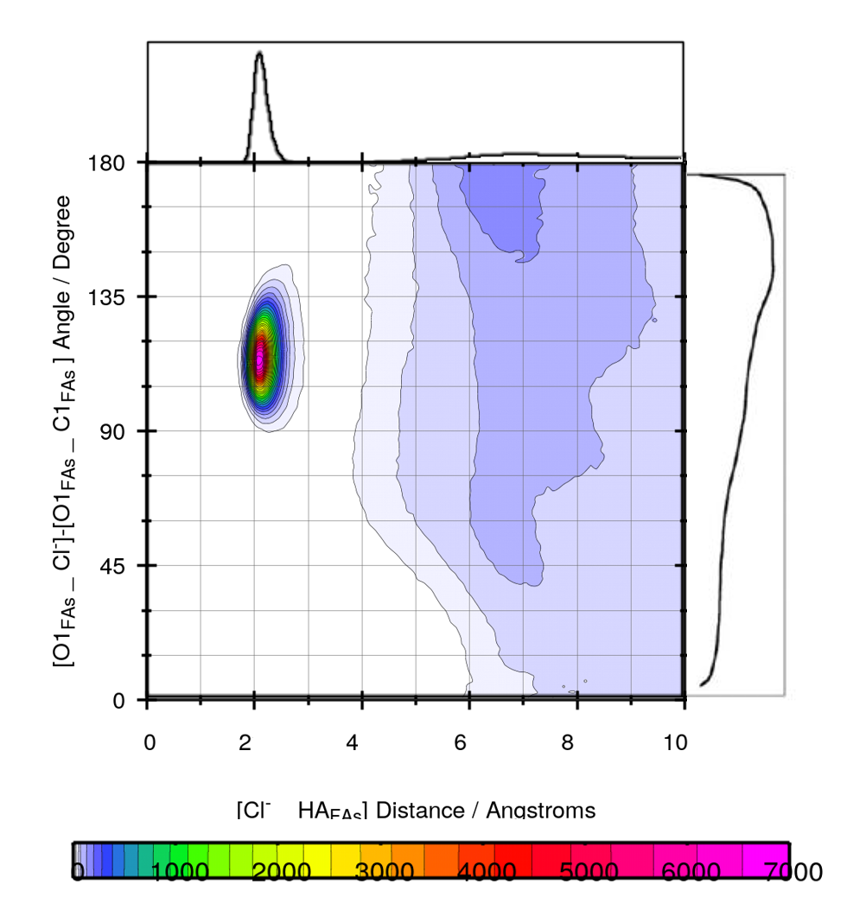


**Fig. S1 (a)**. Combined radial/angular distribution functions for the HA _FAs_ _ Cl^-^ distance and C1_FAs_ – O1 _FAs_ – Cl^-^ angle in the binary mixtures with the mole percent at 30 % of FAs.


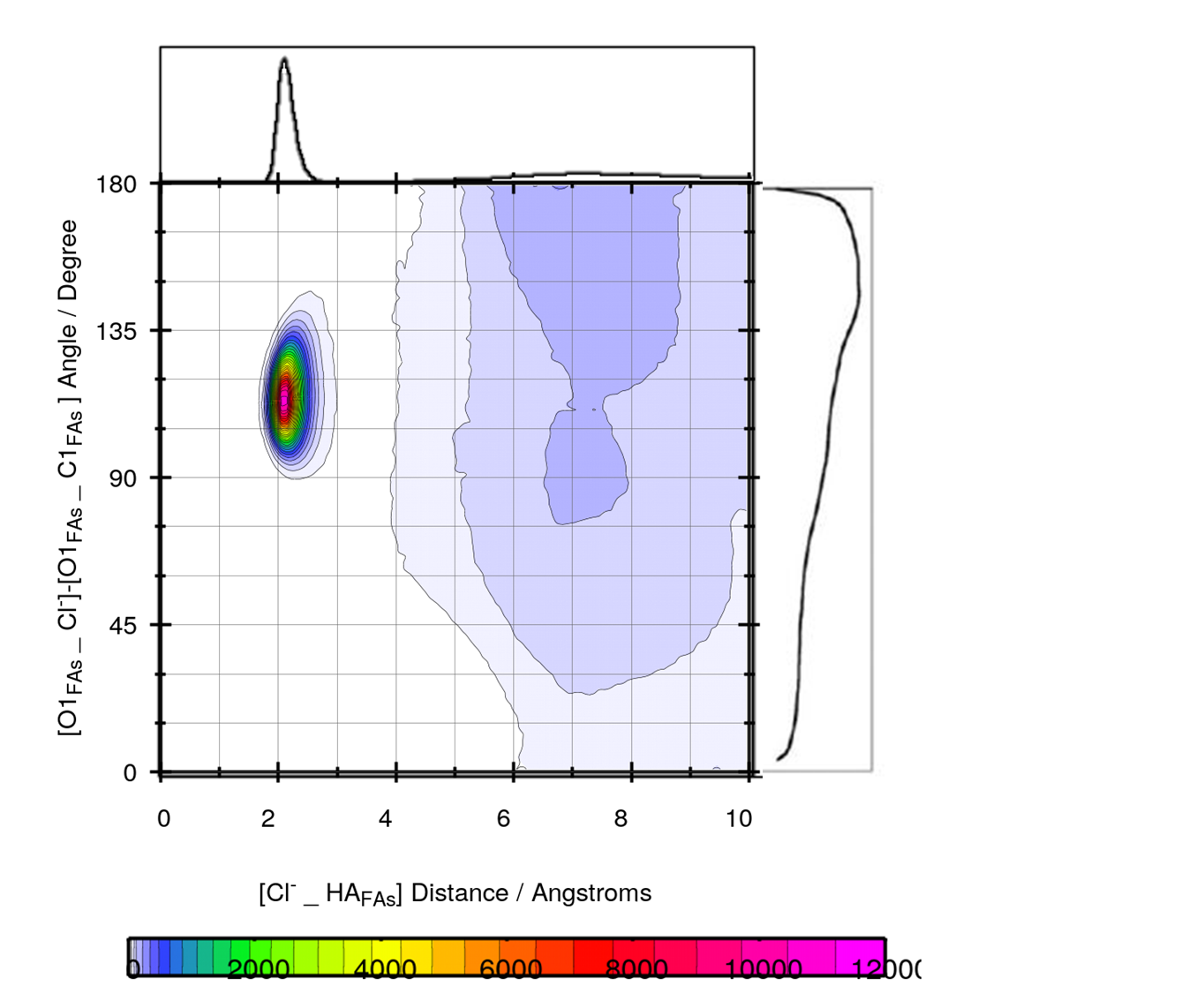


**Fig. S1 (b)**. Combined radial/angular distribution functions for the HA _FAs_ _ Cl^-^ distance and C1_FAs_ – O1 _FAs_ – Cl^-^ angle in the binary mixtures with the mole percent at 50 % of FAs.


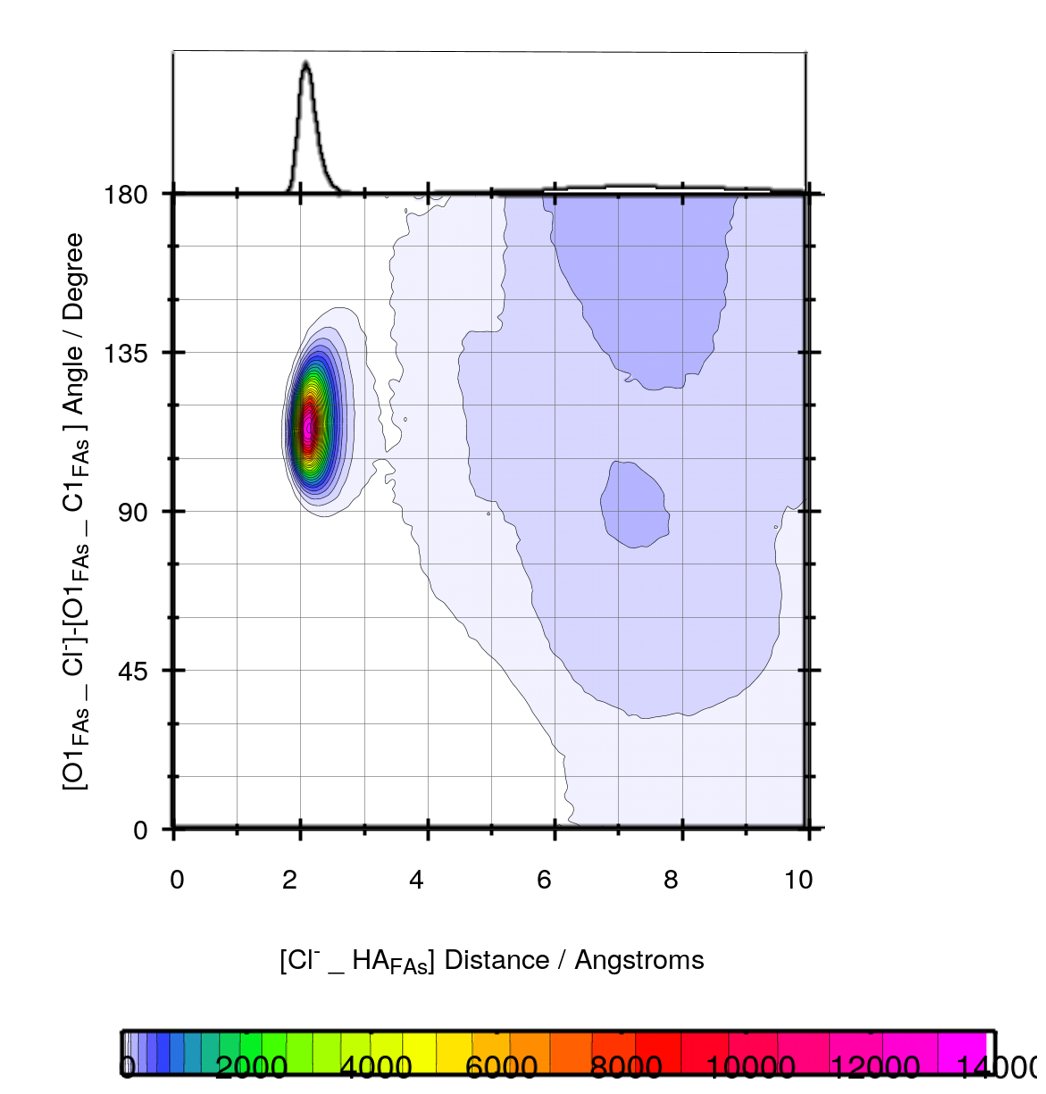

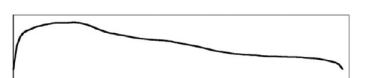


**Fig. S1 (c)**. Combined radial/angular distribution functions for the HA _FAs_ _ Cl^-^ distance and C1_FAs_ – O1 _FAs_ – Cl^-^ angle for the binary mixtures with the mole percent at 70 % of FAs.


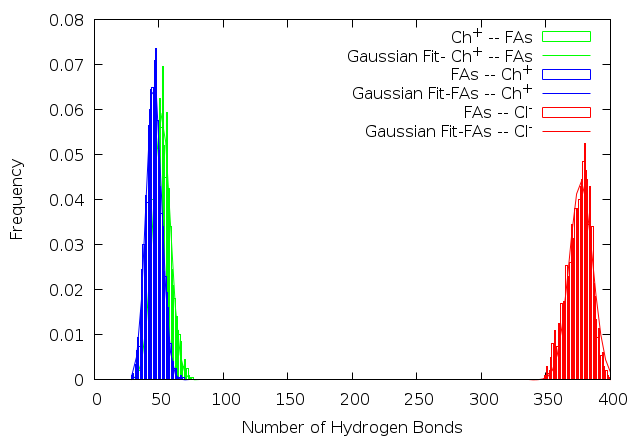


Fig. S2. The distribution of the hydrogen bond between the CAP molecules and [Ch^+^][Cl^-^] Salt in the mixtures containing 50% FAs at 353 K.

**Fig.S3.** The self-diffusion coefficient of chloride anions of DES2 as a function of percentage composition.


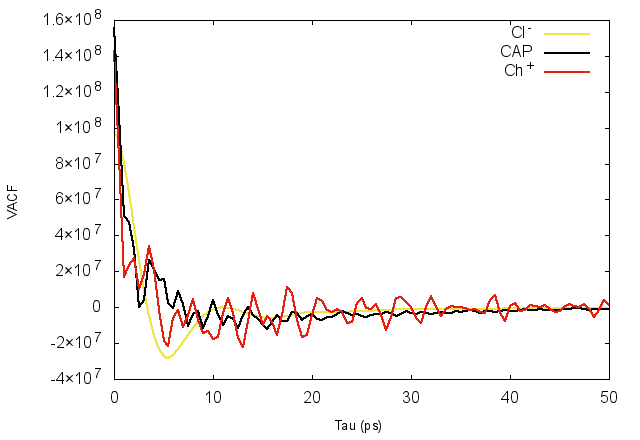


**Fig. S4**. The calculated VACF for the center of mass of [Ch^+^] and CAP molecule and [Cl^-^] anions in the mixture containing 50 % FAs at 353 K.


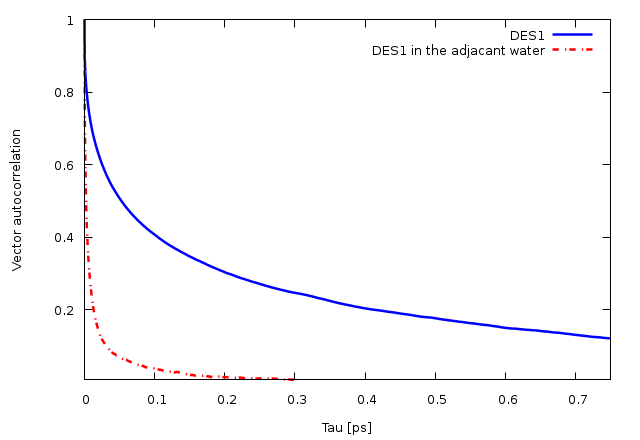


**Fig. S5.** Vector reorientation dynamics for bond (O1--HA) for the pure [Ch^+^/Cl^-^][FAs] DES and the binary mixtures containing 50 % in adjacent water.
